# Axenfeld‐Rieger syndrome combined with a foveal anomaly in a three‐generation family: a case report

**DOI:** 10.1186/s12886-021-01899-2

**Published:** 2021-03-29

**Authors:** Kinga Gołaszewska, Natalia Dub, Emil Saeed, Zofia Mariak, Joanna Konopińska

**Affiliations:** Department of Ophthalmology, Medical University of Bialystok, Jana Kilinskiego 1 STR, 15-089 Białystok, Poland

**Keywords:** Axenfeld‐rieger syndrome, Rieger anomaly, glaucoma, Foveal hypoplasia, Posterior embryotoxon, Corectopia, Hypodontia

## Abstract

**Background:**

Axenfeld-Rieger syndrome (ARS) is a rare autosomal dominant eye disorder that can also affect other organs of the human body. The condition is primarily characterized by the anterior segmental abnormalities of the eye. Here, we present an observational case series of a three-generation family with ARS and unexpected foveal anomaly.

**Case presentation:**

A 33-year-old woman was admitted to an Ophthalmology Clinic in Bialystok for left eye congenital cataract surgery. The patient (proband) was diagnosed with visual deterioration, multiple defects of iris, corectopia, displacement of the Schwalbe’s line, and phenotypic characteristics of ARS. A perimetric examination indicated peripheral visual field loss and signs typical for glaucoma. Based on the phenotypic symptoms and genetic test, the patient was diagnosed with Axenfeld Rieger Syndrome. However, the optical coherence tomography of the macula showed foveal anomaly (absence of the physiological pit), which is not typically associated with this genetic disorder. The patient’s family history revealed that her two daughters were undergoing treatment for congenital glaucoma, and one of the daughters also had foveal anomaly the same as her mother. Interestingly, an examination of the patient’s mother showed typical phenotypic features of ARS such as a defect of the iris, posterior embryotoxon, and coloboma, as well as foveal anomaly. A genetic test confirmed *PITX2* mutation in both, proband’s two daughters and mother.

**Conclusions:**

This study highlights the occurrence of ARS with unusual ophthalmic features such as foveal anomaly (absence of the physiological pit) in a three-generation family. Although ARS is known to represent the developmental defects of the anterior segment of the eye, it is very important to perform fundus evaluation to identify associated posterior segment anomalies that may affect visual acuity. The presence of ocular defects not typically associated with ARS suggests a wide spectrum of mutations within PITX2 gene which are required to identify in order to determine genotype- phenotype correlation in ARS affected individuals.

## Background

Axenfeld-Rieger syndrome (ARS) is an inherited, mostly autosomal dominant developmental disorder, but it could also be sporadic [1]. Clinical features are variable and can be divided into ocular and extra-ocular symptoms. ARS has traditionally encompassed three different subcategories [2] – Axenfeld’s anomaly, which is characterized by the anterior displacement of the Schwalbe’s line, thereby causing posterior embryotoxon; Rieger’s anomaly, which consists of the iris stroma hypoplasia, pupil displacement (corectopia), pigmentation (ectropion uveae), and secondary glaucoma; and Rieger syndrome, which is a combination of Rieger anomalies, and non-ocular malformations such as hypodontia or microdontia, as well as craniofacial abnormalities such as maxillary hypoplasia, broad and flat nasal bridge, telecanthus, and hypertelorism [3]. Around 50 % of patients with Rieger’s anomaly will eventually develop glaucoma in their early childhood or young adulthood. Such cases are often associated with abnormalities in the angle of filtration or secondary angle closure due to adhesions [4]. The diagnosis of ARS can be made by a constellation of ocular findings, including excessive iris tearing (polycoria), iris hypoplasia, eccentric pupils, prominent and displaced Schwalbe′s line (posterior embryotoxon), and iridocorneal tissue adhesions. Extraocular developmental abnormalities, especially of the teeth, facial bones, and periumbilical skin, have also been reported to be associated with ARS [5].

A clear delineation of each of these phenotypes is seldom observed and since most cases present with overlapping features, the term “Axenfeld-Rieger syndrome” is commonly used to refer to such cases [6]. Although ARS-associated characteristics have been well-documented, fundus anomalies, other than typical glaucomatous changes during the clinical course of ARS, have not yet been reported to be associated with ARS. This syndrome is primarily caused by PITX2 and FOXC1 mutations but can also include PAX6 and FOXF1 mutations [7]. Mutations in PITX2 and FOXC1 genes are estimated to account for ∼40 % of ARS cases [8, 9]. To date, 5 chromosomal loci have been implicated to cause ARS – 4q25, 6p25, 11p13, 13q14, 16q24, which include different genes such as *PITX2*, *FOXC1*, *PAX6*, and *FOXF1*. Based on the genetic cause and phenotypic traits, ARS can be categorized into three different types – Type 1, caused by mutations in the *PITX2* gene; Type 2, unknown gene but it is believed to be located on chromosome 13 [9, 10]; and Type 3, caused by mutations in the *FOXC1* gene. Although several studies have reported the occurrence of ARS in different families, only one previous report, to the best of our knowledge, have shown ARS with coexisting atypical foveal anomaly in a three-generation family. The findings of previous studies suggest that the deterioration of the best corrected visual acuity (BCVA) in subjects with ARS is usually caused by anterior segment anomalies such as cataract and/or advanced glaucoma.

In this study, we encountered a unique patient with both typical and atypical ARS features. Since the occurrence of ARS combined with a foveal anomaly is extremely rare, we aimed to understand the molecular basis and inheritance pattern of this phenotype by performing detailed ocular and genetic examinations in both the patient and her three-generation family members.

## Case presentation

This study was performed after obtaining approval from the Bioethics Committee of the Medical University of Bialystok and in accordance with the ethical standards laid down in the 1964 Declaration of Helsinki and its later amendments or comparable ethical standards.

All subjects provided written informed consent for the examination (including extraction of their DNA) and use of their clinical data for publication.

### Case I: 2 (the proband)

A 33-year-old female patient was admitted to the Department of Ophthalmology, Medical University of Bialystok, Poland, in September 2018, for developmental cataract left eye surgery.

**Fig. 1:**
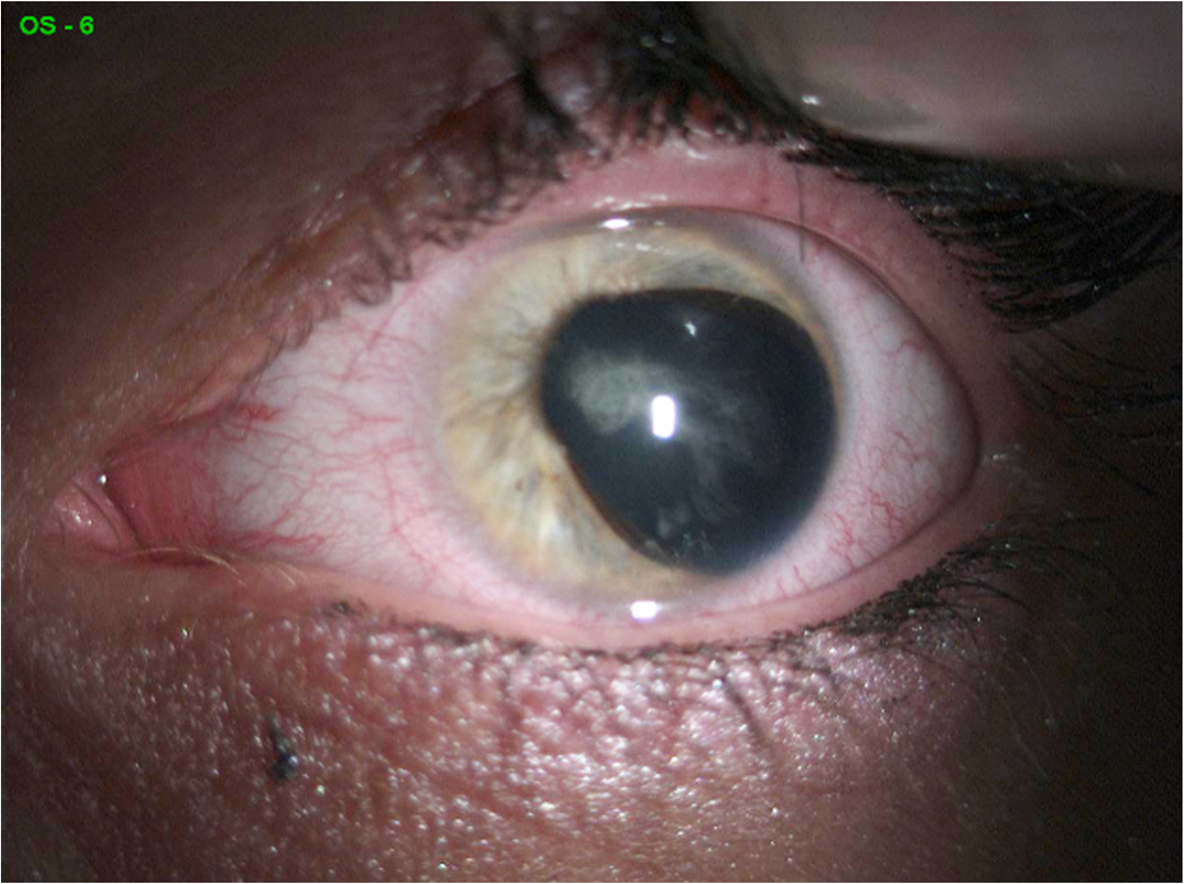
Ocular characteristic of the proband with ARS. Corectopia, left eye

**Fig. 2:**
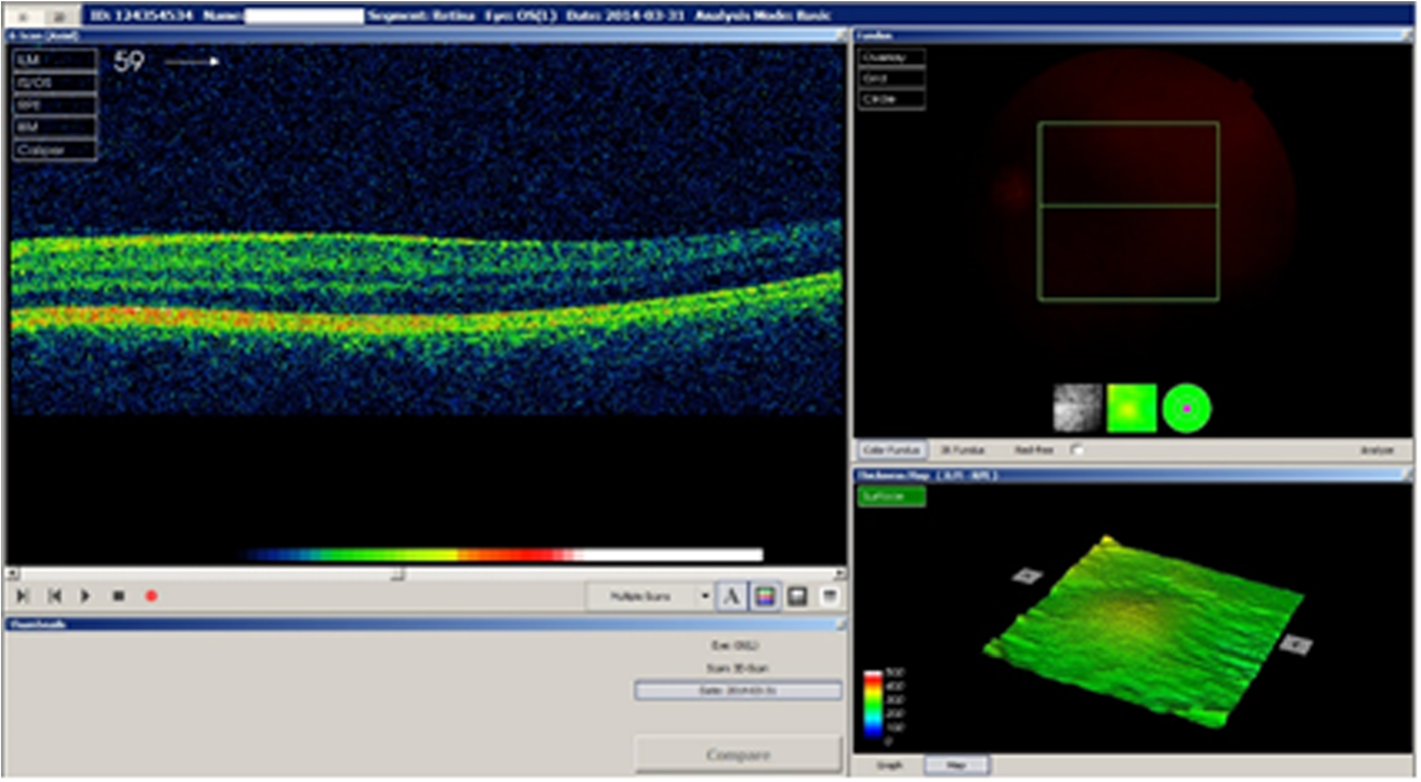
Optical coherent tomography revealed foveal hypoplasia

The proband complained of blurred vision for the past 6 months in both eyes. She did not receive any ophthalmic medications in the past, except 0.5 % timolol 2 times daily for the last 2 years after the diagnosis of glaucoma. We performed a comprehensive ophthalmic examination. The BCVA of both her eyes was 0.5 (Snellen notification converted into decimals, which equals logMAR, 0,3), while the intraocular pressure (IOP, measured by the Goldmann Applanation Tonometer) was 16 and 19 mmHg in the right and left eye, respectively. Slit-lamp microscope examination, anterior segment photography, gonioscopy, fundus examination, field of vision test (Humphrey Visual Field Analyzer, 24 − 2 Carl Zeiss, Jena, Germany) and optical coherence tomography (OCT) measurements of the retina and retinal nerve fibre layer (RNFL) thickness (3D OCT − 1000 Topcon, Tokyo, Japan) were performed. Three-dimensional cube scans centred at the fovea were also obtained. The location of the foveal scan was determined based on the features of foveal specialization as described elsewhere [11]. Slit-lamp examination, in addition to cataract, showed a displacement of the pupil in both eyes – corectopia (Fig. 1), and anteriorly displaced Schwalbe’s line – posterior embryotoxon. Additionally, gonioscopy indicated iridocorneal adhesions. Collectively, these features are indicative of anterior angle dysgenesis in both eyes. Fundus examination showed that the cup-to-disc ratio was 0.5 in both eyes and the macula was without reflection. OCT revealed foveal anomaly, which was characterized by absence of the foveal pit (Fig. 2). Among the perimetry features arcuate scotoma was detected in upper hemisphere (which could be caused by cataract artefact as well) and RNFL thickness measurements revealed nerve fibre defects at the lower quadrants. General examination showed mild dysmorphic facial features – hypertelorism, wide nasal bridge, and hypodontia. Based on these clinical symptoms and phenotypic features, the patient was diagnosed with Axenfeld Rieger Syndrome. We looked for PITX2 mutation that may cause an unusual ophthalmic feature in this family. The screening of the *PITX2* gene for possible mutations was the only available test in Poland at the time of this study. Therefore, a different mutation test for comparison was not available to order. The genetic test was carried out at the GENESIS Medical Genetics Center in Poland. Genomic DNA was extracted from the patient’s blood sample following standard procedures (DNA miniprep kit for blood – Axygen Scientific, Union City, CA, USA) as described elsewhere [12]. Expending genomic DNA from the obtained specimen, the coding regions and splice junctions of the supposed gene were PCR amplified and capillary sequencing is performed (the technique of genetic testing PITX2 gene was Sanger sequencing of the coding exonic and flanking intronic regions of the respective genes.) The bi-directional sequence was collected, aligned to reference gene sequences based on NCBI RefSeq transcript and human genome build GRCh37/UCSC hg19, and examined for sequence variants. Concurrent Multiplex Ligation-dependent Probe Amplification (MLPA) was performed to detect common whole gene copy number events of the evaluated gene(s) in the specimen, compared to control specimen(s). Reported clinically significant variants were confirmed by using alternate primer pairs to significantly reduce the possibility of allele drop-out.

Sequence analysis of the PITX2 gene revealed c.206G > A (P.R69H) nonsense mutation involving chromosome 4q25. This study is the first reported case of this mutation that extends the phenotypic consequences of PITX2 mutations to foveal hypoplasia.

**Fig. 3:**
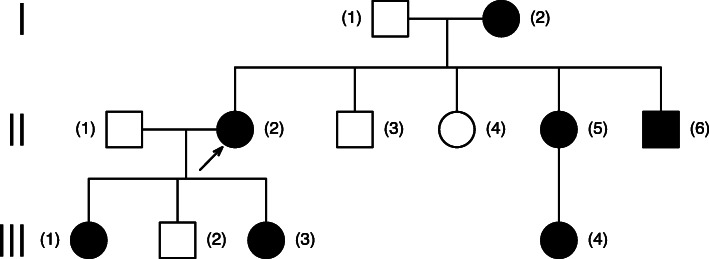
Figure 3. Patient’s pedigree; Legend: (II,2) proband, our patient (c.206G > A (P.R69H) mutation - result of probands genetic testing); (III,1)(III,3) affected daughters (c.206G > A (P.R69H) mutation - information from interview); (II,5) affected sister and her daughter (III,4) (we have only information from interview that location of mutation was on chromosome 4q25) ; (II,6) affected patient’s brother (we have only information from interview that location of mutation was on chromosome 4q25); (I,2) patient’s mother (c.206G > A (P.R69H) mutation - information from interview)

We also examined the history of the patient’s family. Data on other health conditions were collected from the proband and her mother’s medical reports. The patient’s pedigree is shown in Fig. 3.

### Case II: 3 and 4 (the proband’s daughters)

The proband’s 6-year-old and 14-year-old daughters were undergoing treatment for congenital glaucoma in a children’s ophthalmological clinic. They were enrolled in this study in November 2018. Data and ophthalmic medical history were collected, and as mentioned above, both daughters underwent ophthalmological examinations. In the 6-year-old daughter, the BCVA of both eyes was significantly reduced (0.2 in Snellen notification, logMAR 0.7). The IOP, in both eyes, was 18 mmHg (on topical medication timolol 0.5 % applied to both right and left eyes twice daily). Slit-lamp examination revealed iris hypoplasia with cavities mainly temporal and in lower quadrants. Additionally, traces of foetal vessels were found on the lens. Gonioscopy showed iridocorneal adhesions from both angles. Fundus examination revealed that the cup-to-disc ratio was within the normal range. No abnormalities of the macula were perceptible in physical examination. In the 14-year-old daughter, the BCVA of the right eye was 0.9 and that of the left eye was 0.6. The IOP was 15 and 16 mmHg for the right eye and left eye, respectively, after a combined glaucoma therapy (timolol 0.5 % and dorzolamide 2.0 % twice daily one drop to both the right and left eyes). Biomicroscopic examination revealed hypoplasia of the iris parenchyma, ectropion uveae, and lens opacities in both eyes. Gonioscopy showed posterior embryotoxon and a dysgenesis of the angular structure. Fundus examination revealed that the cup-to-disc ratio was within the normal range. OCT examination, performed in both daughters, showed nerve fibre defects. The OCT examination of the macula in the 6-year-old showed, similar to that of her mother, a foveal abnormality, as indicated by the absence of the physiological pit. A general evaluation did not reveal any systemic abnormalities or hearing loss.

### Case III: 1 (the proband’s mother)

The proband’s 54-year-old mother, with a history of cataract surgery in both eyes, was recruited in this study in January 2019. Her BCVA was 0.5 and 0.6 for the right eye and left eye, respectively. She was using a combination of topical medications in both eyes – timolol 0.5 % with dorzolamide 2.0 % (twice daily, one drop in the right and left eye) and latanoprost 0,005 % once daily, (one drop in the right and left eye). Her IOP was 21 and 19 mmHg in the right eye and the left eye, respectively. Slit-lamp examination revealed conveys like iris defects, indicative of posterior embryotoxon. In the fundus examination, the fundal coloboma and a widening of the cup of the optic nerve disc (0.9 cup-to-disc ratio (CDR) in the right eye and 0.8 CDR in the left eye) were observed. Gonioscopy showed multiple iridocorneal adhesions in both eyes. Static perimetry revealed defects in the visual field – arcuate scotoma in upper hemisphere, and the OCT examination of the macula revealed the absence of the physiological pit.

Medical records of the family members did not indicate any history of infantile nystagmus. Ocular albinism such as transillumination defects of the iris and blond fundus were absent.

A genetic examination confirmed a non-sense mutation in the *PITX2* gene in all four subjects, known to occur in ARS. A dental assessment was carried out for the evaluation of craniofacial anomalies and dental abnormalities, which revealed hypodontia, microdontia, and abnormally shaped teeth in all four subjects.

## Discussion and conclusions

ARS, is well characterized by the presence of anterior segment anomalies and typical systemic disorders [13]. In this study, the proband had distinctive ocular signs such as vision deterioration, multiple defects of iris, corectopia, displacement of the Schwalbe’s line indicating posterior embryotoxon, and visual field loss indicative of mild glaucoma. In addition to these typical symptoms that are frequently observed in ARS cases, we also observed foveal anomaly characterized by the absence of physiological pit – a unique finding, which, to the best of our knowledge, has been reported to be associated with ARS only once [10]. Additionally, this unique finding was confirmed in two other members of the proband’s family. Clinical examinations revealed dysmorphic facial features, and dental disorders, while a genetic test confirmed a c.206G > A (P.R69H) nonsense mutation covering chromosome 4q25 in the PITX*2* gene in all four subjects. The PITX2 gene encodes a homeodomain-containing transcription factor, which identifies and binds to specific DNA sequences through the homeodomain, and acts as a transcription regulator during embryogenesis and elaboration of different tissues of the anterior segment. The gene produces four mRNA transcripts (PITX2A-D), but a translation product of PITX2D has never been recognized. The three other isoforms (A–C) differ at the N terminus, but they all include the 60-amino-acid homeodomain. They all have identical C termini with a conserved 14-amino-acid OAR domain (otp, aristaless and rax), which is predicted to mediate protein–protein interactions and self-inhibitory interactions with the N terminus [14].

The integrity of PITX2 is essential for binding DNA and is critical for PITX2 to act as a transcription factor [15]. The above-reported mutation was predicted to abolish the DNA-binding functions of the affected allele and to lead to a premature termination codon (PTC) and are subject to nonsense-mediated mRNA decay (NMD). The mechanism for the PITX2-related severity of the ocular phenotype in these patients may be the consequence of PITX2 haploinsufficiency [14]. This mutation may lead to foveal hypoplasia phenotype.

As mentioned earlier, a foveal anomaly is not typically associated with ARS. This unusual coexistence was previously described by Pal et al. [10] who describe a large consanguineous Asian family with three separate sibships with variations in Axenfeld’s anomaly and posterior embryotoxon, in which foveal hypoplasia was noted following an autosomal recessive pattern of inheritance. They assumed that a defective protein, like PAX6, will have a significant role in the developmental process that controls the formation of the eye.

The absence of foveal pit can usually be observed in cases of hereditary developmental retinal disorders such as albinism, PAX6-related phenotypes, achromatopsia [16], as well as aniridia [17] and Stickler syndrome [18]; infantile nystagmus is a common feature associated with these conditions [16]. However, foveal hypoplasia as an isolated defect, without any other ocular abnormalities, has been previously observed [19].

Previous studies have described some unusual ocular manifestations in ARS cases such as detached Schwalbe’s line suspended in the anterior chamber [20], which can also be explained by impaired neural crest cell migration and differentiation during embryonic development. Different types of esotropia caused by the atypical hypoplasia of different extraocular muscles, which are derived from the mesodermal complex, have also been reported [21]. Interestingly, a previous study reported a posterior segment abnormality in ARS cases such as retinal detachment due to proliferative vitreoretinopathy or due to persistent hyperplastic primary vitreous [22]. However, to the best of our knowledge, only one study to date has described foveal hypoplasia in one patient among a group of twenty-six unrelated patients with different forms of ARS[8]. Similar to the findings of our study, the patient in the above study had also dental and midface abnormalities caused by a *PITX2* gene mutation as well as hearing defects [8].

Several studies have reported the inheritance of ARS. Wu et al. reported a case where two of the 7 living family members were affected by ARS [23]. The authors detected a novel heterozygous mutation in the *FOXC1* gene of the affected family members. Yang et al. reported 5 ARS cases, in a three-generation family, with only ocular manifestations [12]. The authors detected two novel *FOXC1* mutations, and suggested that ocular abnormalities (such posterior embryotoxon, iridocorneal adhesion, iris hypoplasia, or corectopia) and systemic abnormalities are frequently and occasionally, respectively, reported in ARS cases resulting from *FOXC1* mutations; whereas, *PITX2* mutations mainly cause polycoria and systemic abnormalities along with ocular defects. Additionally, *FOXC1* duplication is often characterized by iridogoniodysgenesis, and has a worse prognosis than other mutations [23]. In our study, we did not screen for *FOXC1* mutations for the reason mentioned above. However, it should be noted that in all our patients the course of glaucoma, as well as the visual function, were quite well preserved, including in the oldest affected member of the proband’s family (Case III: 1). Such observations suggest that these patients most likely did not harbour *FOXC1* mutations.

Along with *PITX2* defects, mutations in the *PAX6* gene, located on chromosome 11p13, as well as the deletion of 13q14 and 16q23-24 chromosomal loci, where a causative gene is yet to be identified, have been implicated to cause ARS [24, 25]. Recent studies also showed that mutations in the *CYPB1* gene, which plays a crucial role in vitamin A metabolism and thus, is essential for ocular development, and the *PRDM5* gene, which is responsible for the differentiation and maintenance of extracellular matrix, can contribute to the development of ARS [26–28]. However, despite many advancements in genetic research, the molecular cause in approximately 60 % of ARS cases still remains elusive [28].

This study has a few limitations. First, we only screened for *PITX2* mutations, since it was the only test available in Poland at the time of this study. Second, we did not perform genetic tests in the rest of the family members and data on their health were obtained from interviews. Finally, we did not screen our patients for potential *FOXC1* mutations. PAX6 mutations were also not tested, PITX2 AND PAX6 mutations are unlikely to occur together. Even with the above limitations, the main finding of this study – atypical report of ARS combined with the absence of foveal pit in a three-generation family – is noteworthy.

In summary, our study described a novel presentation of an unusual ophthalmic phenotype in the form of a foveal anomaly (absence of the physiological pit) in a three-generation ARS family with a *PITX2* mutation. The presence of ocular defects, which are not typically associated with ARS, suggests that a wide spectrum of mutations/genetic alterations can contribute towards ARS aetiology. Additional studies are required to validate a causal association between ARS-related mutations and foveal anomaly, as well as to identify other implicated genes .

## Data Availability

The datasets used and/or analysed during the current study are available from the corresponding author on reasonable request.

## Abbreviations

ARS: Axenfeld-Rieger syndrome
BCVA: Best corrected visual acuity
IOP: Intraocular pressure
OCT: Optical coherence tomography
CDR: Cup-to-disc ratio
RNFL: Retinal nerve fibre layer
